# The Progress of Vaccine Treatment

**Published:** 1909-06-12

**Authors:** 


### The Hospital

A Journal of

The Medical Sciences and Hospital Administration.

New Series. No. 120, Vol. V. [No. 11[illegible]2, Vol. XLVI.] SATURDAY, JUNE 12, 1909.

### The Progress of Vaccine Treatment.

Although treatment by vaccines has been actively pursued for some time, we are still far from enjoying precise knowledge of its virtues and limitations. Like all new methods of treatment, it has suffered at the hands of enthusiastic disciples, who have somewhat scared sober minds by the largeness of the claims entered for it. Not that we need complain, since it is humanly impossible for the ardent pioneer in unbroken ground to preserve a strictly detached and judicial attitude towards his work; and it is likely that if he were capable of an absolutely cold judgment, his enterprise in the business would suffer proportionately. Neverthe-less the relations of *post* and *propter* in treatment remain as equivocal as they were when Hippocrates wrote ἡ δὲ πεῖρα σφαλερὴ, ἡ δὲ κρίσις χαλεπὴ, and only patience and perseverance can give us the truth.

For the consummation of this end the periodical collation of unselected cases treated by the new method, with a judgment upon the results made at first hand by practitioners of repute, is of the highest service: and if the judgment be delivered by one not himself exposed to the witchery of the new craft, so much the better. Such a collation has lately been made public in a paper read before the Therapeutical and Pharmacological Section of the Royal Society of Medicine by Drs. Hale White and Eyre. The paper deals with the results of a year's use of vaccines in general medicine, and includes cases of various types. The effects of tuberculin are specifically ex-cluded from this review on the ground that tuber-culosis is, as a rule, so slow a disease that it is impossible to draw conclusions about it within the compass of a twelvemonth. But exclusive of this malady the record of cases is unselected.

The total number of patients was 29, of whom 10 were examples of genito-urinary infection. Four instances of gonorrhœal arthritis were treated by gonorrhœal vaccines with truly remarkable success, the most striking being the following: A woman aged 49 years had suffered from multiple arthritis for nine months, was believed to be the subject of osteoarthritis, and had undergone many methods of treatment in vain. Although the gonococcus could not be isolated from a vaginal discharge to which she was subject, it was determined to administer gono-coccal vaccine on the ground that the opsonic index to this organism was unduly high. Three doses of a stock gonococcal vaccine were therefore given at intervals of seven days. Under this treatment the arthritis rapidly subsided, the patient being dis-charged, cured, within a month. The remaining six genito-urinary cases were examples of infection of the urinary passages by the bacillus coli communis. The most persuasive may again be quoted. A woman aged 45 years had been ailing for five weeks from a multiple arthritis followed at the end of the fourth week by severe cystitis. At the time of her coming under the notice of the authors she was extremely ill, highly febrile, and if not moribund, yet apparently a hopeless case. The bacillus coli communis was isolated from the urine, and a vaccine prepared from her own strain of the organism. A dose of 5,000,000 bacilli was followed by immediate improvement in her symptoms. Two subsequent doses of 30,000,000 and 250,000,000 respectively were given at intervals of a few days and recovery was uninterrupted. Of this case the authors say that "the patient was des-perately ill when seen, and had been ill so long that it appeared to us clear that the vaccine treatment saved her life, and it is of great interest to observe how quickly improvement followed the first injection." The remaining cases of infection of the urinary tract by bacillus coli communis are less dramatic, but four of the six were decidedly benefited, while two showed no response to the treatment. There were suc-cesses, though less striking, in diseases of other organs also.

It will be seen from this summary that we have good reason to be optimistic about the future of vaccine treatment. If it did no more than give us a weapon against an inveterate disease like gonor-rhœal arthritis it would have justified itself, but it appears capable of giving us more. The success so far achieved will at least stimulate research touching the precise origin of a good many diseases not com-monly considered bacterial. The frequency with which the urinary tract becomes infected by bacillus coli communis has only received due appreciation within recent years. It now seems certain that the colon may fall a victim to the same organism, and that even the stomach is liable to its attacks. The feeling that we have at command a possible remedy for these out-of-the-way manifestations of bacterial activity will certainly promote the search for them. All that is required to extract the maximum of benefit from vaccines is the maintenance of a strictly scientific and critical attitude in the weighing of the evidence offered from time to time. Without these precautions vaccines must inevitably share with many other more or less meritorious remedies in the past the undeserved reaction which follows overpraise.

